# Gender-dependent effect of coffee consumption on tremor severity in de novo Parkinson’s disease

**DOI:** 10.1186/s12883-019-1427-y

**Published:** 2019-08-14

**Authors:** Bang-Hoon Cho, Seong-Min Choi, Byeong C. Kim

**Affiliations:** 1 Department of Neurology, Korea University Anam Hospital, Korea University College of Medicine, Seoul, South Korea; 2 Department of Neurology, Chonnam National University Hospital, Chonnam National University Medical School, Gwangju, South Korea; 3 National Research Center for Dementia, Gwangju, South Korea

**Keywords:** Parkinson’s disease, Coffee, Tremor, Gender differences

## Abstract

**Background:**

Coffee consumption represents a negative risk factor for Parkinson’s disease (PD) and seems to affect PD motor symptoms. We aimed to investigate the association between coffee consumption and motor symptoms in de novo PD patients.

**Methods:**

In total, 284 patients with de novo PD were included in the current study. Motor and non-motor symptoms were evaluated using various scales. History of coffee consumption was obtained via a semi-structured interview.

**Results:**

In total, 204 patients were categorized as coffee drinkers and 80 as non-coffee drinkers. Coffee drinkers were predominantly male and had early symptom onset; in addition, they were younger, reported more years in formal education, and had better motor and non-motor scores than did non-coffee drinkers. After adjustments, coffee drinkers had lower tremor scores than did non-coffee drinkers, and coffee consumption was related to tremors in a dose-dependent manner. These relationships were statistically significant in case of rest tremor but not in case of action tremor. The dose-dependent relationship between coffee consumption and tremor severity was significant only in men. Non-motor symptom scores were not significantly different between coffee drinkers and non-coffee drinkers.

**Conclusions:**

Coffee consumption and tremor severity are inversely related in male patients with de novo PD.

## Background

Parkinson’s disease (PD) is a heterogeneous neurodegenerative disorder with diverse clinical manifestations that include both motor and non-motor symptoms [1]. Cardinal motor symptoms used for diagnosing PD include bradykinesia, which is one of the most important features, as well as rigidity, resting tremor, and postural instability [2]. Since tremor may have a different pathophysiology than bradykinesia and rigidity [3], the response of tremor to dopaminergic agents is less clear than that of bradykinesia or rigidity [4].

Previous epidemiological studies established that coffee is a negative risk factor for PD [5–8]. This is further supported by a study showing that caffeine, a major chemical component of coffee, attenuates the loss of striatal dopamine and dopamine transporter binding sites in an experimental PD mouse model [9]. Additional experimental and observational PD studies demonstrated that coffee consumption had a beneficial effect on PD motor symptoms [10, 11]. Since coffee consumption reduces the risk of PD, there might be a relationship between coffee consumption and the severity of motor symptoms. However, few studies have investigated the relationship between coffee consumption and PD motor symptoms.

Gender differences in PD phenotypic presentation and disease progression are widely accepted, and the impact of caffeine on PD differs based on gender [5, 12–15]. In addition, the association between coffee consumption and the severity of motor symptoms may differ between genders. Therefore, in the current study, we investigated the association between coffee consumption and motor symptoms in de novo PD patients based on their gender.

## Methods

### Study population

We performed a baseline survey to determine the association between coffee consumption and motor symptoms in a cohort of newly diagnosed, treatment-naïve, early-stage PD patients consisting of 284 participants. All participants were enrolled from the outpatient clinic at the Chonnam National University Hospital and were recruited consecutively from January 2011 to December 2016.

Parkinsonism comprises multiple syndromes including PD. Thus, according to the international criteria, both parkinsonism and PD symptoms must be evaluated to make a proper PD diagnosis [2]. Parkinsonism is defined by the presence of bradykinesia and one additional symptom, including 4–6 Hz rest tremor, muscular rigidity, or postural instability not caused by primary visual, vestibular, cerebellar, or proprioceptive dysfunction, according to a previous study [2]. The United Kingdom Parkinson’s Disease Society Brain Bank clinical diagnostic criteria were used for the clinical diagnosis of PD [2]. An additional inclusion criterion for our study was the lack of significant cerebral lesions, as assessed by brain magnetic resonance imaging (MRI). None of the patients had a history of taking either antiparkinsonian or antidopaminergic agents. In contrast, the exclusion criteria were an uncertain diagnosis, secondary or atypical parkinsonism according to the recent clinical diagnostic criteria [16–19], failure to complete the coffee consumption questionnaire, and dementia. The time at which one of the four cardinal PD signs was first noted by the patient or caregiver was defined as the onset of PD.

All participants provided written informed consent. The study was approved by the Institutional Review Board of the hospital and was performed in accordance with the ethical standards of the 1964 Declaration of Helsinki.

### Clinical evaluation

All PD patients were diagnosed by a specialist in movement disorders (S.M.Choi). The history of the patient and a neurological examination were used to obtain detailed clinical information, prior to the administration of antiparkinsonian medication.

Participants underwent a thorough neurological examination and were asked about the age at symptom onset and duration of formal education, as well as past and current medications. Both the severity and stage of the parkinsonism were evaluated using the modified Hoehn and Yahr (mHY) staging scale and Unified Parkinson’s Disease Rating Scale (UPDRS) motor (part III) and activities of daily living (ADL; part II) sub-scores. The effect of coffee consumption on the sum of each motor symptom score in the UPDRS, in addition to postural instability and gait disturbance (PIGD) and akinetic-rigid (AR) scores, was evaluated. Patients were scored for these variables using the methods proposed by both Jankovic at al. and Kang et al. [20, 21]. In short, in Jankovic’s method, the tremor score was calculated by dividing the sum of the UPDRS III items 20 and 21 and UPDRS II item 16 by 8, whereas the PIGD score was calculated by dividing the sum of the UPDRS III items 29 and 30 and UPDRS II items 13–15 by 5 [20, 22]. In contrast, in Kang’s method, the tremor score was calculated by dividing the sum of the UPDRS III items 20 and 21 by 4, and the AR score was calculated by dividing the sum of the UPDRS III items 22–27 and 31 by 15 [21].

To evaluate non-motor PD symptoms and general cognition, the Korean versions of the Mini-Mental State Examination (K-MMSE) and the Montreal Cognitive Assessment (MoCA-K) were used [23, 24]. Additionally, the Non-Motor Symptoms assessment scale for PD (NMSS) and the Beck Depression Inventory (BDI) were used [25, 26].

Data on past and present coffee consumption were obtained via a semi-structured interview. In brief, the interview had the following questions: 1) Have you ever had coffee or do you currently drink coffee? 2) If you have ever drunk coffee, when did you start drinking coffee? 3) How long have you been drinking coffee? 4) On average, how many cups of coffee do you drink per day? 5) If you have stopped drinking coffee, when did you stop drinking coffee? For the analyses, we allocated our participants to two groups, the coffee drinker and non-coffee drinker group.

### Statistical analysis

The results are presented as the mean ± standard deviation (SD) for continuous variables while numbers and percentages are provided for categorical variables. Comparisons of the demographic and clinical variables between PD patients with and without a history of drinking coffee were conducted using bivariate analyses (Student’s t-test or chi-square test, depending on the data distribution). Adjustments were made for the variables when bivariate analyses revealed a significant difference between coffee and non-coffee drinkers. Comparison of the severity of motor and non-motor scores between PD patients with and without a history of drinking coffee was conducted using analysis of covariance (ANCOVA), which controlled for the significantly different demographic and clinical variables. We further evaluated the dose-dependent effect of the amount of coffee consumption in a day. Additionally, such analyses were performed in each gender subgroup to investigate the gender differences underlying the association. The *p*-values for the motor score on UPDRS and motor score on subtypes were corrected for by multiple testing using the false discovery rate. All statistical analyses were performed using the SPSS software for Windows (version 22.0, IBM corp., Armonk, NY, USA). A *p*-value < 0.05 was considered statistically significant.

## Results

### Comparison between coffee and non-coffee drinkers

Of the 284 de novo PD patients enrolled (Table 1), a total of 204 patients (71.83%) and 80 patients (28.17%) were categorized as coffee drinkers (coffee drinker group) and non-coffee drinkers (non-coffee drinker group), respectively. The coffee drinker group comprised current coffee drinkers and those who have had coffee in the past but have now stopped. Most of the patients who quit drinking coffee had a history of coffee drinking for more than 10 years. As shown in Table 2, intergroup comparisons revealed that coffee drinkers were younger, included more men, younger in age at cardinal motor symptom onset, and reported more years in formal education than the non-coffee drinkers. When comparing motor- and ADL-related scores, coffee drinkers had significantly lower UPDRS motor scores (*p* = 0.020) as well as tremor (*p* = 0.002), bradykinesia (*p* = 0.022), and gait and posture (*p* = 0.022) scores than non-coffee drinkers. When the tremor was divided into rest and action tremors, only rest tremor (*p* = 0.001) showed a significant difference. Furthermore, the univariate analysis revealed that coffee drinkers had significantly lower tremor scores (*p* = 0.001 for Jankovic’s method, *p* = 0.002 for Kang’s method) than non-coffee drinkers. However, PIGD and AR scores were not significantly different between the two groups. When comparing non-motor scores, we found that coffee drinkers had higher MMSE (*p* = 0.005), but lower BDI (*p* = 0.005) and total NMSS (*p* = 0.007) scores than non-coffee drinkers.

**Table 1:** Demographics and clinical characteristics of patients with Parkinson’s disease (*N* = 284)

Age (years)	65.76 ± 9.63
Sex (female:male)	137:147
Age at symptom onset (years)	63.82 ± 9.69
Duration of disease (months)	21.98 ± 26.13
Formal education (years)	7.77 ± 5.17
Modified H-Y stage	1.79 ± 0.80
UPDRS motor score	20.37 ± 10.65
UPDRS ADL score	7.35 ± 5.63
MMSE	25.49 ± 3.84
MoCA	23.69 ± 4.74
BDI	12.42 ± 9.44
NMSS score	53.27 ± 46.67

The values represent the mean ± standard deviation for continuous variables and numbers for categorical variables

*H-Y* Hoehn and Yahr, *UPDRS* Unified Parkinson’s disease rating scale, *ADL* Activities of daily living, *MMSE* Mini-Mental State Examination, *MoCA* Montreal Cognitive Assessment, *BDI* Beck Depression Inventory, *NMSS* Non-Motor Symptoms assessment scale for Parkinson’s disease

**Table 2:** Comparison of Parkinson’s disease characteristics between coffee drinkers and non-coffee drinkers

	Coffee drinker (*n* = 204)	Non-coffee drinker (*n* = 80)	*p*-value	Adjusted *p*-value
Demographic				
Age (years)	64.57 ± 9.83	68.79 ± 8.24	0.001	
Sex (female:male)	84:120	53:27	< 0.001	
Age at symptom onset (years)	62.84 ± 10.00	67.00 ± 8.15	0.001	
Disease duration (months)	21.08 ± 24.23	22.04 ± 22.65	NS	
Formal education (years)	8.54 ± 5.30	5.77 ± 4.28	< 0.001	
Motor and ADL				
Modified H-Y stage	1.75 ± 0.80	1.89 ± 0.84	NS	
UPDRS motor score	19.46 ± 9.97	22.84 ± 12.04	0.020	NS*
UPDRS ADL score	7.02 ± 5.24	8.22 ± 6.60	NS	
Motor scores on UPDRS				
Tremor	2.48 ± 2.17	3.64 ± 2.90	0.002	< 0.001*†
Rest tremor (Item 20)	1.49 ± 1.67	2.41 ± 2.23	0.001	0.001*†
Action tremor (Item 21)	0.99 ± 1.25	1.23 ± 1.45	NS	NS*
Bradykinesia	8.93 ± 5.36	10.83 ± 6.28	0.022	NS*
Rigidity	4.60 ± 2.83	4.92 ± 2.99	NS	
Gait and Posture	0.78 ± 1.19	1.16 ± 1.27	0.022	NS*
Motor scores on subtypes				
Tremor score (Jankovic)	0.44 ± 0.32	0.61 ± 0.41	0.001	< 0.001*†
PIGD score (Jankovic)	0.41 ± 0.47	0.47 ± 0.52	NS	
Tremor score (Kang)	0.62 ± 0.54	0.91 ± 0.72	0.002	< 0.001*†
Akinetic-Rigid score (Kang)	0.90 ± 0.51	1.05 ± 0.59	NS	
Non-motor symptoms				
MMSE	25.88 ± 3.71	24.38 ± 4.00	0.005	NS**
MoCA	23.99 ± 4.63	22.31 ± 5.04	NS	
BDI	11.32 ± 8.35	15.47 ± 11.51	0.005	NS**
Total NMSS score	48.80 ± 46.12	65.72 ± 46.24	0.007	NS**

NS, statistically not significant

* Adjusted *p*-values were calculated by ANCOVA after adjustment for age, sex, age at symptom onset, formal education period, MMSE, BDI and total NMSS score

** Adjusted *p*-values were calculated by ANCOVA after adjustment for age, sex, age at symptom onset, formal education period, and UPDRS motor score

† Means statistically significant after correcting for multiple testing using the false discovery rate

After adjustment for significant clinical variables found in the univariate analysis, we found that rest tremor score (*p* = 0.001) on UPDRS and tremor scores on Jankovic’s and Kang’s methods (all *p*-values < 0.001) were lower in coffee drinkers than in non-coffee drinkers. However, scores related to other motor manifestations, such as bradykinesia, gait and posture, PIGD, and AR, were not significantly different between the two groups. Furthermore, after adjustment, there was no significant difference between the two groups for all the non-motor symptom scores (Table 2).

Since the prevalence of coffee consumption differed according to gender, we compared the demographic and clinical variables between coffee drinkers and non-coffee drinkers in each gender. Similar to the results described above, rest tremor scores were lower in coffee drinkers than in non-coffee drinkers in both the male and female subgroups, after adjusting for the significant variables found in the univariate analysis (Table 3).

**Table 3:** Comparison of Parkinson’s disease characteristics between coffee drinkers and non-coffee drinkers across genders

	Male				Female			
	Coffee drinker (*n* = 120)	Non-coffee drinker (*n* = 27)	*p*-value	Adjusted *p*-value	Coffee drinker (*n* = 84)	Non-coffee drinker (*n* = 53)	*p*-value	Adjusted *p*-value
Demographic								
Age (years)	64.36 ± 10.04	72.20 ± 6.05	< 0.001		65.01 ± 9.45	67.32 ± 8.82	NS	
Age at symptom onset (years)	62.63 ± 10.14	69.75 ± 6.24	< 0.001		63.29 ± 9.71	65.53 ± 8.62	NS	
Disease duration (months)	21.00 ± 26.51	29.36 ± 43.61	NS		21.21 ± 20.69	22.02 ± 21.93	NS	
Formal education (years)	10.15 ± 5.18	7.74 ± 4.66	NS		6.16 ± 4.50	4.94 ± 3.80	NS	
Motor and ADL								
Modified H-Y stage	1.75 ± 0.74	2.00 ± 0.78	NS		1.72 ± 0.83	1.82 ± 0.86	NS	
UPDRS motor score	19.47 ± 8.50	24.13 ± 11.69	0.024	NS*	18.99 ± 11.26	22.22 ± 12.27	NS	
UPDRS ADL score	7.15 ± 5.24	8.84 ± 6.03	NS		6.66 ± 5.17	7.98 ± 6.86	NS	
Motor scores on UPDRS								
Tremor	2.52 ± 2.42	4.28 ± 3.46	0.021	0.002*†	2.44 ± 1.76	3.32 ± 2.55	0.018	0.036*
Rest tremor (Item 20)	1.43 ± 1.83	2.84 ± 2.66	0.017	0.004*	1.57 ± 1.41	2.20 ± 1.98	0.034	0.044*
Action tremor (Item 21)	1.08 ± 1.28	1.08 ± 1.44	NS	NS*	0.86 ± 1.20	1.12 ± 1.38	NS	NS*
Bradykinesia	8.60 ± 4.48	10.72 ± 5.82	0.043	NS*	9.40 ± 6.40	10.88 ± 6.56	NS	
Rigidity	4.71 ± 2.70	5.00 ± 3.30	NS		4.44 ± 3.00	4.88 ± 2.85	NS	
Gait and Posture	0.68 ± 1.01	1.20 ± 1.00	0.020	NS*	0.93 ± 1.40	1.14 ± 1.40	NS	
Motor scores on subtypes								
Tremor score (Jankovic)	0.45 ± 0.35	0.68 ± 0.48	0.006	0.005*†	0.42 ± 0.27	0.58 ± 0.37	0.007	0.026*†
PIGD score (Jankovic)	0.38 ± 0.45	0.45 ± 0.48	NS		0.46 ± 0.49	0.48 ± 0.54	NS	
Tremor score (Kang)	0.63 ± 0.61	1.07 ± 0.86	0.021	0.002*†	0.61 ± 0.44	0.83 ± 0.64	0.018	0.036*†
Akinetic-Rigid score (Kang)	0.89 ± 0.44	1.05 ± 0.57	NS		0.92 ± 0.60	1.05 ± 0.60	NS	
Non-motor symptoms								
MMSE	26.41 ± 3.47	25.67 ± 3.13	NS		25.11 ± 3.92	23.79 ± 4.24	NS	
MoCA	24.20 ± 4.03	23.18 ± 4.35	NS		23.55 ± 5.49	21.87 ± 5.51	NS	
BDI	10.43 ± 7.91	11.04 ± 9.08	NS		12.56 ± 8.83	17.68 ± 12.02	0.010	0.018**
Total NMSS score	52.26 ± 51.36	59.76 ± 39.25	NS		43.95 ± 37.30	68.70 ± 49.47	0.003	0.009**

NS, statistically not significant

* Adjusted *p*-values were calculated by ANCOVA after adjustment for age, age at symptom onset, formal education period, MMSE, BDI and total NMSS score

** Adjusted *p*-values were calculated by ANCOVA after adjustment for age, age at symptom onset, formal education period, and UPDRS motor score

† Means statistically significant after correcting for multiple testing using the false discovery rate

### Dose-dependent relationship between coffee consumption and PD symptoms

The median value of coffee consumption was one cup a day; therefore, PD patients were categorized as non-coffee drinkers, coffee drinkers - one cup a day, and coffee drinkers - more than one cup a day, to evaluate the dose-dependent effect of the coffee consumption on tremor. We found that mHY stage, UPDRS motor, UPDRS tremor, UPDRS rest tremor, UPDRS bradykinesia, UPDRS gait and posture, tremor scores in both the Jankovic’s and Kang’s methods, and the AR score in Kang’s method were inversely related to the number of cups of coffee per day, and this was significant. Additionally, we found a significant inverse relationship between the BDI score and the number of cups of coffee per day, although MMSE and MoCA scores were directly related to the number of cups of coffee per day. However, after adjustment for the confounders, only tremor scores (UPDRS tremor, UPDRS rest tremor, and tremor scores in both Jankovic’s and Kang’s methods) were found to be related to coffee consumption in a dose-dependent manner (Table 4).

**Table 4:** Comparisons of demographic and clinical characteristics among Parkinson’s disease patients categorized by coffee drinking status (Non-coffee drinker, Coffee drinker - 1 cup a day, Coffee drinker - more than 1 cup a day [median = 1])

	Non-coffee drinker (*n* = 80)	Coffee drinker 1 cup (*n* = 99)	Coffee drinker more than 1 cup (*n* = 105)	*p*-value	*P* for trend	Adjusted *p*-value
Demographic						
Age (years)	68.79 ± 8.24	66.52 ± 8.97	62.52 ± 10.22	< 0.001	< 0.001	
Sex (female:male)	53:27	48:51	36:69	< 0.001	< 0.001	
Age at symptom onset (years)	67.00 ± 8.15	64.85 ± 8.94	60.80 ± 10.56	< 0.001	< 0.001	
Disease duration (months)	22.04 ± 22.65	20.40 ± 19.16	20.93 ± 27.00	NS	NS	
Formal education (years)	5.77 ± 4.28	8.15 ± 4.86	8.92 ± 5.53	< 0.001	< 0.001	
Motor and ADL						
Modified H-Y stage	1.89 ± 0.84	1.82 ± 0.72	1.66 ± 0.82	NS	0.028	NS*
UPDRS motor score	22.84 ± 12.04	20.9 ± 9.6	17.9 ± 9.9	0.006	0.002	NS*
UPDRS ADL score	8.22 ± 6.60	7.0 ± 5.5	6.8 ± 5.2	NS	NS	
Motor scores on UPDRS						
Tremor	3.64 ± 2.90	2.56 ± 2.37	2.40 ± 2.07	0.003	0.001	0.002*†
Rest tremor (Item 20)	2.41 ± 2.23	1.77 ± 1.43	1.41 ± 1.73	0.001	< 0.001	0.002*†
Action tremor (Item 21)	1.23 ± 1.45	1.30 ± 1.40	0.90 ± 1.20	0.076	0.074	NS*
Bradykinesia	10.83 ± 6.28	9.61 ± 5.72	8.89 ± 5.22	0.037	0.012	NS*
Rigidity	4.92 ± 2.99	5.13 ± 2.96	4.28 ± 2.75	NS	NS	
Gait and Posture	1.16 ± 1.27	0.90 ± 1.07	0.68 ± 1.27	0.015	0.004	NS*
Motor scores on subtypes						
Tremor score (Jankovic)	0.60 ± 0.41	0.45 ± 0.35	0.43 ± 0.28	0.004	0.001	0.003*†
PIGD score (Jankovic)	0.47 ± 0.52	0.43 ± 0.47	0.38 ± 0.50	NS	NS	
Tremor score (Kang)	0.89 ± 0.72	0.64 ± 0.59	0.60 ± 0.50	0.003	0.001	0.002*†
Akinetic-Rigid score (Kang)	1.04 ± 0.60	0.95 ± 0.52	0.85 ± 0.48	NS	0.018	NS*
Non-motor symptoms						
MMSE	24.38 ± 4.00	25.3 ± 3.7	26.2 ± 3.7	0.009	< 0.001	NS**
MoCA	22.31 ± 5.04	22.7 ± 5.2	25.1 ± 3.6	0.001	0.001	NS**
BDI	15.47 ± 11.51	11.66 ± 7.90	11.00 ± 8.84	0.006	0.003	NS**
Total NMSS score	65.72 ± 46.24	48.25 ± 47.25	50.48 ± 45.88	NS	NS	

* Adjusted *p*-values were calculated by ANCOVA after adjustment for age, sex, age at symptoms onset, formal education period, MMSE, MoCA, and BDI

** Adjusted *p*-values were calculated by ANCOVA after adjustment for age, sex, age at symptom onset, formal education period, and UPDRS motor score

† Means statistically significant after correcting for multiple testing using the false discovery rate

Finally, we evaluated the dose-dependent effect of coffee consumption on tremor in each gender. Tremor scores (UPDRS tremor, UPDRS rest tremor, and tremor scores in both the Jankovic’s and Kang’s methods) had an inverse relationship with coffee consumption in a dose-dependent manner only in the male subgroup, after adjustment for significant clinical variables found in the univariate analysis (Table 5).

**Table 5:** Comparisons of demographic and clinical characteristics among Parkinson’s disease patients categorized by coffee drinking status between genders (Non-coffee drinker, Coffee drinker - 1 cup a day, Coffee drinker - more than 1 cup a day [median = 1])

	Male						Female					
	Non-coffee drinker (*n* = 27)	Coffee drinker 1 cup (*n* = 51)	Coffee drinker more than 1 cup (*n* = 69)	*p*-value	p for trend	Adjusted *p*-value	Non-coffee drinker (*n* = 53)	Coffee drinker 1 cup (*n* = 48)	Coffee drinker more than 1 cup (*n* = 36)	*p*-value	*p* for trend	Adjusted *p*-value
Demographic												
Age (years)	72.04 ± 6.00	67.29 ± 9.08	62.03 ± 10.26	< 0.001	< 0.001		67.62 ± 8.68	65.69 ± 8.89	63.47 ± 10.22	NS	0.038	
Age at symptom onset (years)	69.68 ± 6.17	65.72 ± 8.78	60.17 ± 10.54	< 0.001	< 0.001		65.69 ± 8.51	63.94 ± 9.11	62.01 ± 10.63	NS	0.070	
Disease duration (months)	28.30 ± 42.08	18.98 ± 20.12	22.69 ± 30.81	NS	NS		23.79 ± 25.26	21.98 ± 18.13	17.61 ± 17.65	NS	NS	
Formal education (years)	8.11 ± 4.88	9.78 ± 5.27	10.31 ± 5.14	NS	NS		4.73 ± 3.85	6.61 ± 3.89	5.93 ± 5.23	NS	NS	
Motor and ADL												
Modified H-Y stage	1.93 ± 0.79	1.94 ± 0.73	1.64 ± 0.72	NS	NS		1.84 ± 0.86	1.70 ± 0.70	1.71 ± 0.98	NS	NS	
UPDRS motor score	23.18 ± 11.71	22.28 ± 9.12	17.78 ± 7.60	0.007	0.010	NS*	22.40 ± 12.27	19.50 ± 10.47	17.80 ± 12.08	NS	NS	
UPDRS ADL score	8.67 ± 5.86	7.63 ± 5.77	6.81 ± 4.88	NS	NS		8.21 ± 6.72	6.27 ± 4.82	6.72 ± 5.64	NS	NS	
Motor scores on UPDRS												
Tremor	4.15 ± 3.36	2.55 ± 2.82	2.49 ± 2.14	0.017	0.006	0.014*†	3.26 ± 2.57	2.58 ± 1.75	2.25 ± 1.70	NS	0.026	NS*
Rest tremor (Item 20)	2.84 ± 2.66	1.62 ± 1.47	1.39 ± 1.92	0.006	0.001	0.016*†	2.20 ± 1.98	1.95 ± 1.40	1.45 ± 1.41	NS	0.017	NS*
Action tremor (Item 21)	1.44 ± 1.58	1.46 ± 1.50	0.98 ± 1.21	NS	NS		1.12 ± 1.38	1.10 ± 1.26	0.79 ± 1.18	NS	NS	
Bradykinesia	10.26 ± 5.85	8.96 ± 5.10	8.45 ± 3.99	NS	NS		11.00 ± 6.65	9.49 ± 6.03	8.97 ± 6.73	NS	NS	
Rigidity	4.81 ± 3.27	5.42 ± 3.10	4.25 ± 2.26	NS	NS		4.91 ± 2.99	4.63 ± 2.68	4.11 ± 3.21	NS	NS	
Gait and Posture	1.15 ± 0.99	0.88 ± 1.13	0.54 ± 0.90	0.018	0.008	NS*	1.21 ± 1.45	0.85 ± 1.13	0.92 ± 1.63	NS	NS	
Motor scores on subtypes												
Tremor score (Jankovic)	0.66 ± 0.46	0.46 ± 0.41	0.44 ± 0.30	0.032	0.011	0.027*	0.57 ± 0.38	0.44 ± 0.28	0.41 ± 0.25	0.035	0.020	NS*
PIGD score (Jankovic)	0.42 ± 0.47	0.44 ± 0.52	0.34 ± 0.40	NS	NS		0.49 ± 0.55	0.43 ± 0.43	0.48 ± 0.56	NS	NS	
Tremor score (Kang)	1.04 ± 0.84	0.64 ± 0.71	0.63 ± 0.53	0.017	0.006	0.014*†	0.82 ± 0.64	0.65 ± 0.44	0.56 ± 0.42	NS	0.026	NS*
Akinetic-Rigid score (Kang)	1.00 ± 0.57	0.96 ± 0.51	0.85 ± 0.37	NS	NS		1.06 ± 0.61	0.94 ± 0.54	0.87 ± 0.64	NS	0.147	
Non-motor symptoms												
MMSE	25.80 ± 3.14	25.88 ± 3.80	26.76 ± 3.20	NS	NS		23.78 ± 4.16	25.12 ± 3.32	25.24 ± 4.69	NS	NS	
MoCA	23.22 ± 4.22	23.34 ± 4.68	24.82 ± 3.44	NS	NS		21.58 ± 5.57	22.26 ± 5.59	25.62 ± 4.07	0.016	0.009	NS**
BDI	10.82 ± 8.83	10.80 ± 7.65	10.23 ± 8.22	NS	NS		17.45 ± 11.84	12.56 ± 8.14	12.47 ± 9.88	0.024	0.025	NS**
Total NMSS score	57.41 ± 39.26	53.61 ± 58.40	51.97 ± 46.36	NS	NS		66.08 ± 49.24	42.56 ± 31.05	47.61 ± 45.45	0.017	0.047	0.044**

NS, statistically not significant

* Adjusted *p*-values were calculated by ANCOVA after adjustment for age, age at symptoms onset, formal education period, MMSE, MoCA, and BDI

** Adjusted *p*-values were calculated by ANCOVA after adjustment for age, age at symptom onset, formal education period, and UPDRS motor score

† Means statistically significant after correcting for multiple testing using the false discovery rate

## Discussion

This study investigated the relationship between coffee consumption and motor symptoms in de novo PD patients. In summary, we found that 1) coffee drinkers have lower tremor scores than non-coffee drinkers, 2) the low tremor scores in coffee drinkers are found in both the male and female subgroups, 3) coffee consumption is related to tremor in a dose-dependent manner, 4) the relationship between coffee consumption and tremor was statistically significant only in rest tremor, not in action tremor, and 5) the dose-dependent, inverse relationship between coffee consumption and tremor scores was significant only in the male subgroup.

The relationship between coffee consumption and tremor is controversial. Some people have previously stated that drinking coffee made their hands prone to tremor, and a small proportion of patients with essential tremor or PD thought that coffee worsened their tremor [27]. However, caffeine only infrequently induces tremor in the general population, and it does not exacerbate pathological tremor [27, 28]. Although caffeine consumption is not correlated to tremor severity in patients with essential tremor [29], currently, no studies exist on the association between caffeine and tremor in PD patients. Our study shows that coffee consumption could attenuate tremor severity in de novo PD patients.

Tremor may have distinct pathophysiology compared to bradykinesia and rigidity. Previous neuroimaging studies reported that patients with the tremor-dominant PD subtype show higher dopamine transporter binding than those with the AR subtype, whereas tremor severity does not correlate to striatal dopamine deficits [30, 31]. Furthermore, another neuroimaging study revealed the involvement of both the cerebellothalamic circuit and basal ganglia in PD tremor generation [32]. Coffee is a major source of caffeine, which is the most widely consumed methylxanthine (1,3,4-trimethylxanthine) and is also a nonspecific adenosine A1/A2A receptor antagonist. Several studies have suggested that A2A receptor antagonists have a tremolytic effect in PD animal models [10, 33, 34]. Although it is possible that caffeine has a symptomatic benefit on tremor, there might be other explanations, including the neuroprotective effect of caffeine, reverse causality (prodromal PD patients might have reduced desires for caffeine), or residual confounding by other factors (specific personality or changes in reward mechanism in PD) [35]. In this study, we found that coffee consumption prior to diagnosis could influence tremor severity in de novo PD patients. Selective adenosine A2A antagonism through the modulation of basal ganglia function via opposing dopamine D2 receptors seems to be effective for the treatment of PD motor symptoms as a monotherapy or an adjunct therapy to dopaminergic agents [36, 37]. We believe that the effects of coffee on tremor severity in PD patients could be caused by the modulation of dopaminergic signaling even before clinical diagnosis. As such, future large prospective studies should investigate the therapeutic effects of coffee or adenosine A2A antagonists on tremor in PD. The findings from this study, therefore, suggest a potential therapeutic option for PD tremor, which responds less effectively to dopaminergic treatment than bradykinesia or rigidity.

In our study, the relationship between coffee consumption and tremor was significant only in rest tremor. One possible explanation is that rest tremor rather than action tremor is a typical symptom that reflects Parkinson’s pathophysiology [38]. However, these results could be related to the limitations of the UPDRS scoring system, because the total score of rest tremor is higher than that of action tremor.

A number of studies on the gender differences in PD patients were reported [13]. For example, although female PD patients show a mild disease phenotype at first, they have a more aggressive disease progression and a higher probability of motor complications as the disease progresses [13]. The impact of coffee and caffeine on PD risk and mortality also differs between genders [5, 12, 14, 15]. In fact, our study shows that the dose-dependent inverse association between coffee consumption and tremor severity is prominent in men but not in women. Such gender differences in PD have been partly explained by the effect of estrogen. Similar to our results, caffeine was reported to reduce the risk of PD, and its beneficial effect was prevented by the use of estrogen replacement therapy [12]. In addition, caffeine was found to reduce the risk of PD in postmenopausal women without hormonal replacement therapy; however, increased PD risk was observed in estrogen users [39]. Since estrogen has neuroprotective or neurotrophic effects and modulates the nigrostriatal dopaminergic system [40], we suggest that estrogen modulates the effects of caffeine on the dopaminergic system and that a complex interaction between caffeine, estrogen, and dopamine exists in the basal ganglia system. Large prospective studies could elucidate the mechanism behind such observations.

In this study, we included de novo PD patients to ensure that the effects of medication would not represent a confounder. Additionally, we prospectively recruited patients with PD based on a registry to reduce the selection bias. Our study has several limitations. First, there were significant differences between coffee drinkers and non-coffee drinkers in demography, including age, sex, age at symptom onset, and formal education. Although, we adjusted for these variables in other analyses, it is possible that these differences could affect the relationship between coffee consumption and tremor. Second, we investigated the association between coffee consumption and motor symptoms in de novo PD patients only, without examining control or advanced PD groups. Further controlled studies would reveal a more precise association between tremor severity and coffee consumption in PD patients. Third, this study did not include information regarding the consumption of other caffeine-containing beverages. There is a possibility that other beverages containing caffeine can influence motor symptoms in PD patients. Fourth, we did not collect information on the menopausal status and hormonal replacement therapy in women. Therefore, the analysis of the association between coffee consumption and motor severity in the female subgroup according to the hormonal status was not possible.

## Conclusion

Coffee drinkers had lower tremor scores when compared to non-coffee drinkers, and the coffee consumption was inversely related to tremor severity in a dose-dependent manner in de novo PD patients. These relationships were statistically significant only in rest tremor, not in action tremor. The effect of coffee consumption on tremor severity was gender-dependent, and it was significant only in men. Further investigations are needed to reveal the exact causal relationship between coffee consumption and tremor in PD patients.

## Data Availability

The datasets used and/or analyzed during the current study are available from the corresponding author on reasonable request.

## Abbreviations

ADL: Activities of daily living
ANCOVA: Analysis of covariance
AR: Akinetic-rigid
BDI: Beck Depression Inventory
K-MMSE: Korean versions of the Mini-Mental State Examination
mHY: modified Hoehn and Yahr scale
MoCA-K: Montreal Cognitive Assessment
MRI: Magnetic resonance imaging
NMSS: Non-Motor Symptoms assessment scale for Parkinson’s disease
PD: Parkinson’s disease
PIGD: Postural instability and gait disturbance
SD: Standard deviation
UPDRS: Unified Parkinson’s Disease Rating Scale
